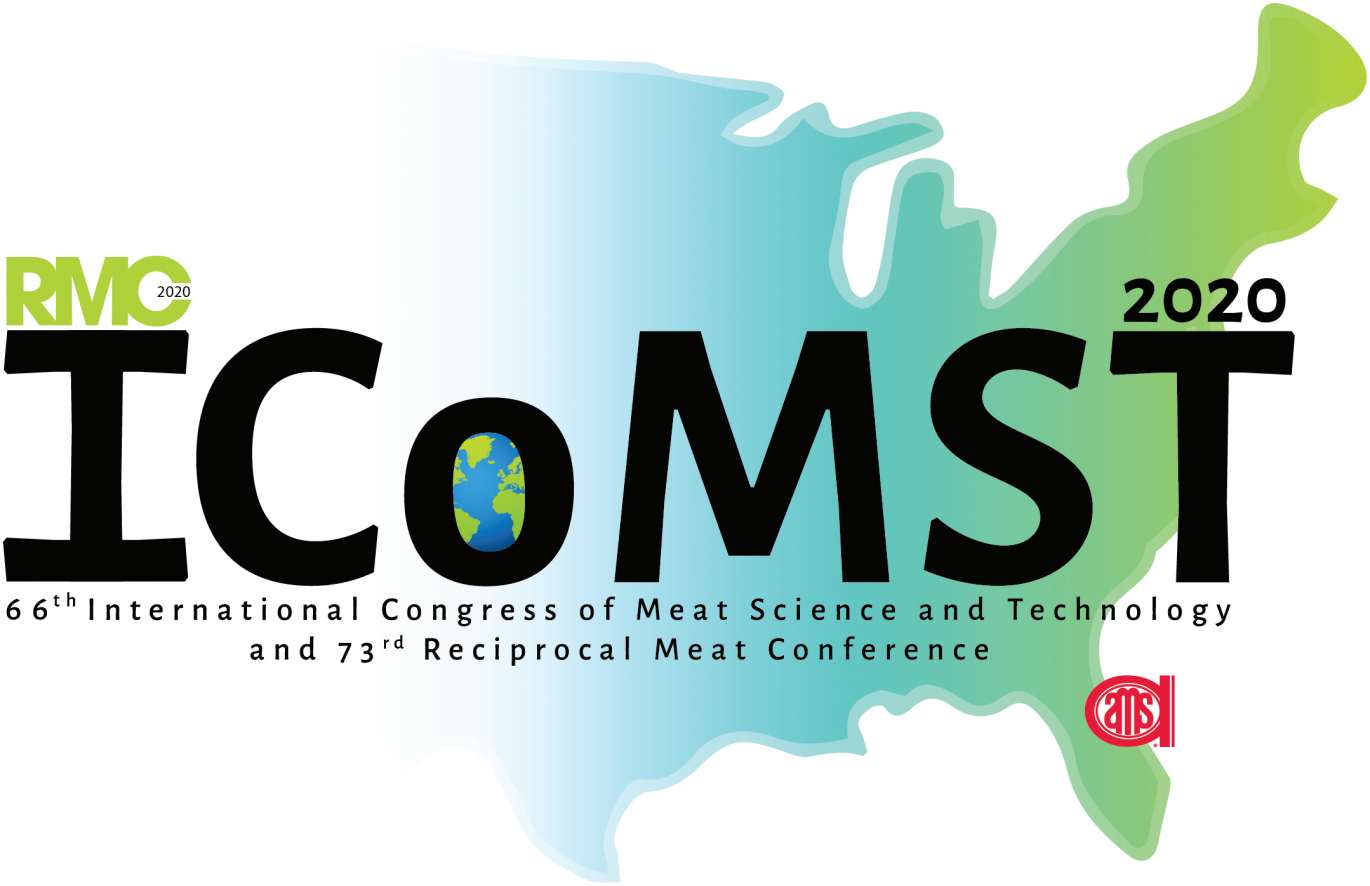# American Meat Science Association News

**DOI:** 10.1093/af/vfz044

**Published:** 2020-01-10

The American Meat Science Association (AMSA) fosters community and professional development among individuals who create and apply science to efficiently provide safe and high-quality meat.

Please Join Us for the 66^th^ ICoMST and 73^rd^ RMC – August 2–7, 2020

In 2020, the American Meat Science Association (AMSA) will welcome the world’s meat science community to the International Congress of Meat Science and Technology and Reciprocal Meat Conference (ICoMST/RMC). It has been 15 years since AMSA has hosted this conference. The 1,200+ attendees from 60 countries will join us in the most magical place on earth, Walt Disney World (Orlando, Florida), to discuss current research and development, food safety, quality assurance and other aspects of meat production from industry, academia and allied organizations.

The event will include six days of intensive technical and educational sessions. The event will kick-off with a headliner presentation address from Pierre Ferrari, CEO Heifer International, focusing on Global Nutrition Security. As well as a look at the future of science and innovation from Jack Uldrich, Global Futurist and Best-Selling Author. The conference will include a presentation focusing on professional development from Dr. Ronnie Green, University of Nebraska Chancellor. A deep dive into corporate responsibility with Jeff Simmons (President and CEO Elanco Animal Health) and Donnie Smith (retired Tyson Foods CEO). Another session that all will wish to attend will focus on animal welfare with the world’s most recognized expert, Dr. Temple Grandin (Colorado State University). There will also be breakout sessions focusing on the science of meat production and processing.

Attendees will also enjoy world class social events and industry tours. Wednesday of the conference will be dedicated to tours allowing attendees to explore all that Florida has to offer. Production facilities such as: Dessert Ranch a world class cattle ranch in central Florida, Adena Springs a Thoroughbred horse breeding facility, an alligator farm, an orange grove and much more. Additionally, there will be opportunities to visit beautiful St. Augustine, NASA space center, and any of the Disney parks.

Finally, there will be over 500 scientific digital poster presentations on cutting edge research, and culinary events that take advantage of the unique venue, and world class social events.

This is a once-in-a-generation opportunity to attend, participate and sponsor this unique conference of ICoMST and RMC together. We hope to see everyone at Walt Disney World in August 2–7, 2020!

For more information please visit www.icomst2020.com or send an email to information@meatscience.org.